# PEGylated Adenoviruses: From Mice to Monkeys

**DOI:** 10.3390/v2020468

**Published:** 2010-02-01

**Authors:** Piyanuch Wonganan, Maria A. Croyle

**Affiliations:** 1 Division of Pharmaceutics, College of Pharmacy, The University of Texas at Austin, Austin, TX 78712, USA; E-Mail: piyanuch@mail.utexas.edu; 2 Institute of Cellular and Molecular Biology, The University of Texas at Austin, Austin, TX 78712, USA

**Keywords:** adenovirus, gene therapy, vaccine, toxicity, PEGylation, targeting, pharmacokinetics, immune response, tolerance, non-viral vectors

## Abstract

Covalent modification with polyethylene glycol (PEG), a non-toxic polymer used in food, cosmetic and pharmaceutical preparations for over 60 years, can profoundly influence the pharmacokinetic, pharmacologic and toxciologic profile of protein and peptide-based therapeutics. This review summarizes the history of PEGylation and PEG chemistry and highlights the value of this technology in the context of the design and development of recombinant viruses for gene transfer, vaccination and diagnostic purposes. Specific emphasis is placed on the application of this technology to the adenovirus, the most potent viral vector with the most highly characterized toxicity profile to date, in several animal models.

## 1. Introduction

Polyethylene glycol (PEG) is one of the most versatile polymers synthesized. This amphiphilic, chemically inert polymer consists of repeating units of ethylene oxide, which can be arranged in either linear or branched configurations, creating a series of compounds of different molecular weights with unique properties [1]. A demonstrated lack of immunogenicity, toxicity and antigenicity, subsequent approval by the United States Food and Drug Administration (U.S. FDA) for human use and high solubility in water and many organic solvents fostered the use of PEG as a key excipient in many pharmaceutical and cosmetic formulations for over 60 years [2]. Other unique properties of this molecule such as flexibility of the polymer chain led to the pioneering work initiated in the late 1970s by Abuchowski and others in the field of molecular modification of biological molecules [3]. Since then, covalent attachment of PEG to protein and peptide-based therapeutics, a technique known as PEGylation, has become one of the most widely applied strategies for improving the physicochemical and pharmacokinetic properties of these labile compounds [4–6]. PEGylation has been used to ameliorate the stability, solubility, bioavailability and immunological properties of many biological compounds such as such as lipids, polysaccharides, polynucleotides and most recently, complex living organisms such as cells, tissues and viruses [3,7–9].

## 2. PEGylation: The Early Years

### 2.1. The Chemistry

**Figure 1.**
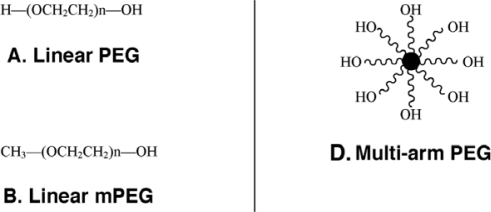

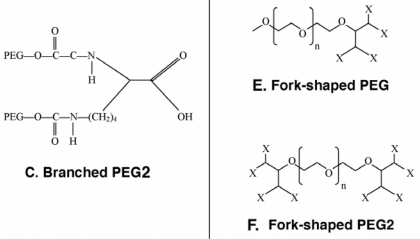
Representative Types of PEG for Protein and Peptide Modification. **(A)** The core molecule, linear poly(ethylene) glycol, a diol, with two free hydroxyl groups; **(B)** Monomethoxy poly(ethylene) glycol (mPEG). The single hydroxyl group is the site of attachment for a variety of reactive groups suitable for conjugation to nucleophilic functional groups on proteins such as lysine; **(C)** A branched PEG molecule in which two linear mPEGs activated with either succinimidyl carbonate or benzotriazole are linked to the ∝- and ɛ-amino groups of lysine. These molecules offer the advantage of adding two PEG molecules at each attachment site, affording broader protection from proteolysis and the immune response without reducing bioactivity; **(D)** Multi-arm PEG. These compounds, generally prepared with hexaglycerine at the core, offer multiple hydroxyl groups for attachment of many copies of the same or several different reactive groups for protein and peptide conjugation; **(E)** Forked shaped PEG. Fork-shaped PEGs provide multiple reactive groups in close proximity at one or both ends (Panel **F**) of the PEG chain, where X represents functional groups.

**Figure 2.**
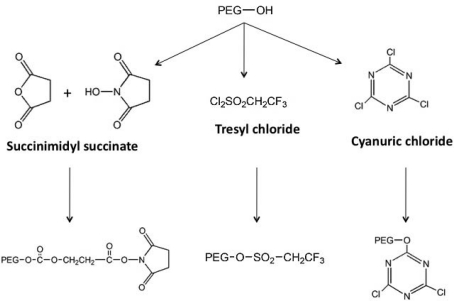
General Schematic for the Synthesis of Activated PEG Molecules.

The monomethoxylated form of PEG (mPEG), containing a single hydroxyl group for activation and an inert methoxy group, resistant to standard chemical reactions, is generally used for protein conjugation (Figure 1)[8]. The first step in the PEGylation process is activation of the PEG molecule prior to protein conjugation. Activation is achieved by substitution of the single hydroxyl group at the end of the PEG molecule by an electrophilic reactive group that can then be covalently linked to a reactive site on a protein (Figure 2). Succinimidyl succinate PEG is prepared by reaction of mPEG with succinic anhydride followed by conversion of the carboxylic acid to the succinimidyl ester. This linkage is highly susceptible to hydrolysis after the polymer has been attached to the protein. Reaction of mPEG with tresyl chloride produces an activated PEG that has been shown to randomly modify proteins, viruses and liposomes, resulting in a heterogeneous mixture of conjugates with degradable linkages. Original PEGylation schemes employed cyanuric chloride to prepare activated PEG for attachment to proteins through multiple nucleophilic functional groups such as lysine, serine, tyrosine, cysteine, and histidine, which supports significant crosslinking and aggregate formation.

**Figure 3.**
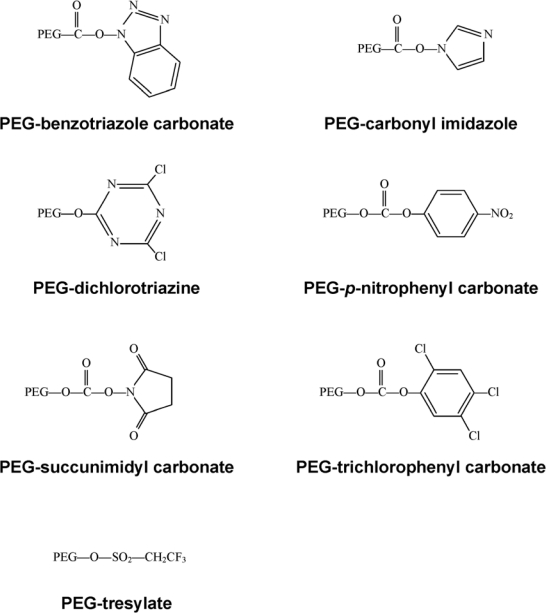
First Generation PEG Derivatives.

Early on, lysine, one of the most plentiful amino acids present in many proteins, was identified as the primary site for attachment of PEG residues and could be modified with a wide selection of chemical moieties (Figure 3) [10], commonly used for PEGylation of both free ∝- and ɛ-amino groups on peptides and proteins. Protocols employing these molecules are often plagued with the presence of impurities and non-specific attachment to residues, allowing for inconsistencies between production lots. These chemistries are also restricted to low molecular weight polymers. Once attached to a bioactive molecule, they are relatively unstable and tend to degrade soon after administration *in vivo*.

**Figure 4.**
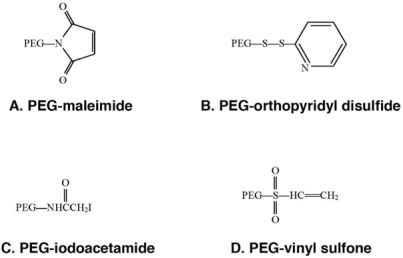
Second Generation PEG Derivatives. These PEG derivatives have been developed for specific attachment to cysteine residues on proteins and peptides. **(A)** PEG-maleimide is able to conjugate to free thiols under acidic (pH 6–7) conditions, however, this compound is not stable in water and the ring is susceptible to opening or addition of water across the double bond. **(B)** PEG-orthopyridyl disulfide reacts specifically with sulfhydryl groups under both acidic and basic conditions (pH 3–10) to form a disulfide bond with proteins which are stable except in a reducing environment when the linkage is converted to a thiol. **(C)** PEG-iodoacetamide, reacts slowly with thiol residues to form stable thioether bonds in mildly basic media. Use of this compound is advantageous in the context that, by strong acid hydrolysis, modification of a protein with this compound gives rise to a stable cysteine conjugate, carboxymethylcysteine that can be identified and quantified by standard amino acid sequencing techniques to verify the degree of modification of the parent protein. It is also important to note that any reaction employing this polymer should be performed under dark conditions in order to prevent the production of free iodine that may react with other amino acids such as tyrosine. **(D)** PEG-vinyl sulfone reacts slowly with thiols to form a stable thioester linkage to proteins under slightly basic conditions (pH 7–8). This process will proceed at a faster rate if the pH is increased. However, under these conditions, PEG-vinyl sulfone may also react with free lysines. Use of any of these compounds is dictated by protein solubility/stability under reaction conditions defined by the PEGylation chemistry, availability/accessibility of cysteine residues on the protein surface, desired speed of reaction and availability of methodology for characterization of protein conjugates.

Improved second generation PEG chemistries have minimized diol contamination and expanded the chemical repertoire to include high molecular weight compounds with very diverse structures (Figure 4) [11].

**Figure 5.**
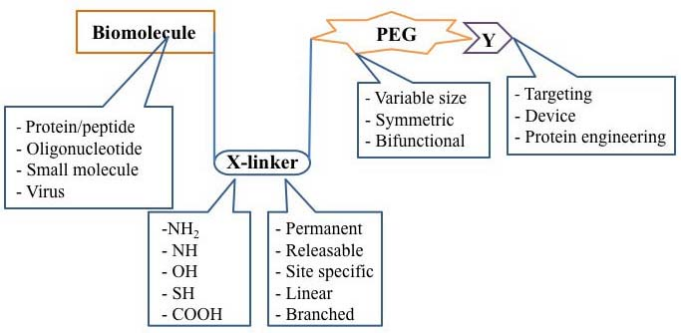
The Building Blocks of a PEGylated Therapeutic. Once a therapeutic molecule amenable to PEGylation is identified, the appropriate PEG chemistry must be selected. Properties such as size, symmetry, and bifunctionality must be considered and adopted according to the desired application. The linker used for covalent attachment of PEG must also be evaluated with respect to the strength of the bond created as well as its affinity for certain residues on the bioactive molecule. Receptor-specific peptides, proteins and molecular sensors have been tethered to PEGylated therapeutics for cellular and tissue specific targeting.

Today, PEGylation processes focus on select amino acids, such as thiol groups on cysteine residues, as primary sites for attachment, thereby minimizing random attachment of PEG to the protein surface and the amount of heterogeneous conjugates in a given preparation [12]. Improved site-specific PEGylation with these reagents minimizes loss of biological activity through enhanced preservation of the native protein structure. Complex, branched second generation PEG molecules have also been shown to maintain pH and thermal stability, reduce immunogenicity and protect modified proteins from proteolysis to a much higher degree than those modified with first generation chemistries [4,5]. Further refinement of these molecules led to the development of heterofunctional PEGs that directly link the protein of interest to another molecule used for targeting specific cells and tissues. A general schematic of PEGylation strategies is summarized in Figure 5.

### 2.2. Tolerization to Antigens, Tissues and Cells

Davis and Abuchowski were the first to modify two model proteins, bovine serum albumin and liver catalase, with methoxypolyethylene glycols (mPEG) of 1,900 and 5,000 Daltons (Da) using cyanuric chloride and 2,4,6-trichloro-8-triazine as the coupling agents, respectively [13,14]. One of their prime findings, that when PEG is properly coupled to a protein or an enzyme, the modified product loses its immunogenicity *in vivo,* laid the groundwork for additional studies that suggested that protein antigens modified by this technique may not only fail to induce an immune response but may be tolerogenic. This phenomenon was first reported by Lee *et al.* when intravenous administration of PEGylated ovalbumin (PEG-OVA) 4 hours before sensitizing with dinitrophenylated OVA (DNP-OVA) suppressed the primary anti-DNP-OVA and anti-OVA IgE responses in both mice and rats. These animals also responded very poorly to additional doses of DNP-OVA. In contrast, injection of unmodified OVA did not alter the ability of the animals to mount IgE responses to DNP-OVA [15]. Additional reports have been written summarizing similar transitions for other allergens including ragweed pollen extract, uricase, l-asparaginase, hen egg lysozyme, and bovine gamma globulin and other antibodies to non-immunogenic, tolerogenic derivatives through PEGylation [16–25].

Although it has been shown that repeat administration of each of these PEGylated allergens significantly reduces IgM, IgG and IgE antibody levels against the native antigen, it has also been found that the degree of suppression depends on the immunological state at the time of treatment, the nature of allergen and the dose. Suppression of the antibody-mediated response was best observed in mice without measurable anti-allergen antibodies, while the weakest suppression was observed in mice with high levels of antibodies at the time of treatment [26]. Additional studies have found that PEGylated allergens can induce a long-term suppression of the antibody response [15,18,21,26,27]. For example, administration of PEGylated human monoclonal immunoglobulins (HIgG) suppressed the anti-HIgG antibody response by more than 95% for over 300 days after a series of seven injections of the immunizing antigen in mice [21]. While the exact mechanism by which this phenomenon occurs remains unknown, adoptive transfer of splenocytes from animals given several doses of PEGylated compounds to naïve animals suggests that PEG-modified allergens activate antigen-specific CD8^+^ suppressor T cells [15,18,27,28]. Since proliferation of suppressor T cells and the associated production of factors that dampen the immune response is dependent upon the concentration of circulating antigen during the tolerization process, slowing of antigen processing and extending the half-life of the antigen by PEGylation most likely plays a major role in the immunosuppressive capacity of the conjugates [17,24,25].

As data continued to suggest that PEGylated biomolecules were less immunogenic than their native counterparts, many interested in preventing immunorejection during transplantation began to apply this process to intact, viable cells and tissues. Modification of type A and type B human red blood cells (RBCs) with mPEG did not compromise the structure, function and viability of the cell and prevented agglutination by anti-A and anti-B antisera, respectively [29–31]. PEGylation of ovine RBCs prolonged their survival when transfused into mice [30]. PEGylation of lymphocytes can inhibit MHC class II activation and proliferation of T cells, as was demonstrated in an *in vivo* model of transfusion-associated graft *versus* host disease [31–33]. Adoptive transfer of PEGylated splenocytes isolated from C57BL/6 mice to lethally irradiated Balb/c mice extended survival time from 7 to 10 days [34]. PEGylation reduced proliferation in the donor T cell population, suggesting that the loss of T cell responsiveness is likely due to disruption interactions between the T cells and antigen presenting cells necessary to mount an immune response [32]. It has also been suggested that this weak, ineffective co-stimulation of alloreactive T cells induces apoptosis, leading to tolerance of donor tissue [34]. This phenomenon has been further exemplified in diabetic rat models where PEGylation improved pancreatic islet viability, facilitated engraftment and re-established blood glucose homeostasis [34,35]. Additional studies demonstrated that PEGylation efficiently blocked recognition of the transplants by immune cells, allowing them to remain stably functional in diabetic recipients for several weeks [35–38].

## 3. PEGylation in the Pharmaceutical Industry

Approval of Adagen® (Pegademase) for the treatment of severe combined immunodeficiency disease (SCID) by the U.S. FDA in the early 1990s illustrated the potential for PEGylation to significantly impact modern therapeutics. The half-life of this product, bovine adenosine deaminase randomly modified with 5 kDa PEG molecules, increased from less than 30 min to 28 hr for the conjugated counterpart [39]. This, along with the fact that the modified enzyme preparation could evoke 1,800 times the activity per milliliter of red blood cells alone [40], marked notable progress in the area of enzyme replacement therapy by minimizing the number of doses needed to achieve a therapeutic effect, eliminating the need for blood transfusion and avoiding the risk of iron overload and transfusion-associated viral infection [11]. In a similar manner, therapeutic use of l-asparaginase for acute lymphoblastic leukemia was hindered by the fact that frequent intramuscular injections, necessary for therapeutic efficacy, also induced strong hypersensitivity reactions, rendering the enzyme ineffective in many patients [41]. Covalent attachment of 5 kDa PEG to this enzyme four years later reduced its immunogenicity and the necessity for frequent dosing, allowing for successful use in those with hypersensitivity to the unmodified enzyme [42–44]. Within the last 10 years, improvements in polymer homogeneity and PEGylation chemistry have produced many potent, well-characterized, protein-based therapeutics. Therapeutic PEGylated proteins currently marketed in the United States and Europe are summarized in Table 1.

Two marketed products of PEGylated interferon alpha (IFN-∝), PEGIntron (Schering Plough) and PEGASYS (Roche), illustrate how different PEG chemistries can affect the pharmacokinetic profile of a biologically active protein. PEGIntron (IFN-∝2b) contains a single chain 12kDa PEG attached to His34 via a urethane bond [45]. Although this modification significantly extends the elimination half-life and reduces the clearance of the protein (Table 2), the conjugate is hydrolytically unstable in plasma and when stored at ambient temperatures [46]. The activity of this preparation relative to the unmodified protein is 28% [47]. In contrast, PEGASYS (IFN∝-2a) contains branched 40 kDa PEG molecules attached to lysine residues via an amide bond [48]. This conjugate, with greater *in vitro* and *in vivo* stability has a significantly longer half life than PEGIntron and reduces the clearance of the protein by a factor of 100, overshadowing the fact that the modified protein retains only 7% of the relative activity of the unmodified protein (Table 2) [47]. Despite their differences, both products have greater anti-viral action than their native counterparts, reducing administration schedules from three times a week to once weekly [49,50].

Granulocyte-colony stimulating factor (G-CSF) and an anti-TNF-∝ Fab′ have been conjugated to PEG in order to increase the overall size of the molecule. In the case of Pegfilgrastim (Neublasta®:PEGylated G-CSF), the increase in size minimizes renal clearance of the drug, prolongs the circulation time and stimulates the proliferation and differentiation of neutrophils which, in turn, aid in clearing the compound once therapeutic levels are attained [51]. Certolizumab pegol (Cimzia®), the Fab′ fragment of a humanized anti-tumor necrosis factor-α (TNFα) monoclonal antibody linked to a branched 40 kDa PEG maleimide at a single cysteine residue, demonstrates a prolonged circulating time in blood, such that a single monthly subcutaneous injection is sufficient for therapeutic efficacy. In addition, increasing the size of this molecule by PEGylation allows it to preferentially accumulate in inflamed tissue, where its therapeutic effect is primarily needed [46].

## 4. PEGylation and Gene Therapy in the 21st Century

Gene therapy has gained significant attention over the past two decades as a potential method for diverse applications including the correction of inherited genetic and neurodegenerative disorders, as well as the treatment of cancer, cardiovascular and infectious diseases [52,53]. Despite encouraging pre-clinical results, practical use of this technology has been hindered by inefficient delivery to specific cellular targets and immune responses to both the vector selected for gene transfer and the transgene product. Rapid clearance from the circulation by the complement system and the immunogenicity associated with non-viral and viral vectors alike were the primary reasons for applying PEGylation technology to gene delivery vectors. In this regard, PEGylation was initially used for non-viral vectors and eventually for viral vectors as the immune response became the most significant limitation to therapeutic gene transfer. Additional efforts soon recognized that advanced PEG chemistries accommodated attachment of specific ligands to the vectors, making them less promiscuous and promoting tissue and cell-specific gene expression.

### 4.1. Non-Viral Vectors

Non-viral gene transfer is a method by which DNA is formulated with cationic lipids and/or polymers to create dense particles that are delivered to target cells by physical means such as direct injection, electroporation or gene gun [54]. These particles, however, are often rapidly sequestered in the reticuloendothelial system (RES), and are removed from the bloodstream within minutes after systemic administration [55,56]. PEGylation was primarily used to reduce the rate at which these vectors are cleared to improve therapeutic efficacy. This has been achieved by PEGylating preparations containing branched polyethylenenimine/β-cyclodextrin/DNA polyplexes as well as simple DNA/chitosan mixtures [57–59]. In both cases, this modification minimized aggregate formation under physiological conditions and prolonged circulation time, thereby reducing uptake by macrophages and improving overall transduction efficiency of each system. Numerous reports have shown that PEGylation also reduces binding of DNA complexes to plasma proteins and minimizes toxicity while improving the stability, solubility and, in turn, transfection efficiency of these non-viral preparations [52,57,60–65]. Results from these studies and data accumulated from clinical trials with viral vectors soon prompted development of PEGylation strategies for viruses.

### 4.2. Viral Vectors

Although viral vectors, unlike their non-viral counterparts, are extremely efficient at inducing transgene expression in cellular targets, their clinical utility is hindered by significant immunogenicity and toxicity. Covalent modification of proteins contained in the virus coat with PEG has been shown to improve both the physicochemical and biological properties of several recombinant viruses used for gene transfer (Table 3) including adeno-associated virus (AAV) [66,67], pseudotyped lentivirus [68], retrovirus [69,70], and baculovirus [71].

PEGylation has also been used to modify influenza virosomes, virus-like particles synthesized from capsid proteins, to minimize host immunological responses [72,73]. While results with many viral vectors have been minimal, there has been a significant effort in developing PEGylation protocols for one of the most potent vectors with a highly characterized toxicity profile to date, the adenovirus.

### 4.3. The Adenovirus

Despite the fact that recombinant adenoviruses can infect both dividing and non-dividing cells, have the capacity to accommodate large DNA inserts and can be readily produced in large quantities with high purity [74], their clinical use is significantly hindered by strong innate and adaptive immune responses against viral proteins [75]. This is compounded by the fact that the virus enters cells through several receptors (integrins, heparan sulfate proteoglycans and the coxsackie- and adenovirus receptor (CAR)) distributed widely throughout the body which leads to non-specific transgene expression and undesired side effects such as thrombocytopenia, intense periportal polymorphonuclear lymphocyte infiltration and elevated liver enzymes [76–78]. Thus, a significant effort was put forth to develop PEGylation protocols for the adenovirus, an otherwise highly efficient vector for gene transfer.

PEGylation of adenovirus was initially achieved by the use of monofunctional PEGs (mPEGs) such as monomethoxypolyethylene glycol activated by tresyl chloride (TMPEG), succinimidyl succinate (SSPEG) and cyanuric chloride (CCPEG) which react with the ɛ-amino terminal of lysine residues on virus capsid proteins (Figure 2) [79–82]. These early studies revealed that modification with TMPEG did not compromise virus titer during a 3 hour reaction period, while modification with SSPEG reduced titer by 30% [80]. Preparations modified with the CCPEG chemistry retained only 11% of their original titer after the 90 minute conjugation process was complete. It was later found that this dramatic loss of titer was due to extensive cross-linking of the polymer with multiple virus capsids and subsequent formation large aggregates. Despite the initial loss of infectious titer during the PEGylation process, each of these preparations maintained titers that were significantly higher than unmodified virus during storage at −20, 4, 25 and 42 °C for extended periods of time. PEGylation of adenovirus prior to microencapsulation with poly(lactic-co-glycolic acid) (PLGA) block copolymers prevented inactivation of the virus during the microencapsulation process, resulting in improved transfection efficiency with respect to unmodified virus used in the same process [83]. Further investigation revealed that this modification prevented virus aggregation within the acidic environment created during degradation of the microspheres and prevented shear-induced damage to the virus capsid during the homogenization process.

Although early studies reported that PEGylation increased transduction by a factor of 3 in the lung [81] and by a factor of 5 in the liver [82], it was also found that excessive PEGylation could critically damage the structure and function of the virus, limiting transduction efficiency [79,84,85]. For example, an increase in the a viral amine:mPEG molar ratio from 1:1 to 1:10 reduced the relative transduction efficiency of the virus from 94% to 52% [83]. Additional studies revealed that transduction fell by 20, 60 and 99% as 25, 50 and 80% of the free amines of the virus are modified with PEG, respectively [84].

### 4.4. Characterization of PEGylated Adenoviruses

Once it was realized that the number of PEG molecules attached to capsid proteins profoundly affects the performance of the virus *in vitro* and *in vivo*, much effort was put forth to develop assays to characterize the physical properties of these vectors and accurately determine the degree of modification that occurred within a given preparation. As PEG molecules are added to the virus surface, they mask protein residues that dictate the overall charge and relative solubility of the particle while increasing the hydrodynamic radius in accordance with the size of PEG. Thus, initial characterization profiles of PEGylated adenoviruses included assays designed to assess broad changes in the physical properties of the virus such as particle size, zeta potential and PEG-Dextran partition coefficients [80,86,87]. Zeta potential analysis revealed that PEGylation reduces the negative charge of the virus capsid from −48 mV (unmodified, native virus) to −28, −24 and −16 mV when conjugated to TMPEG, SSPEG and CCPEG, respectively [80]. The partition coefficient of the PEG-virus conjugate in an aqueous PEG/dextran two-phase system (K) was found to shift from 0.7 for unmodified virus to 1.76, 1.96 and 3.56 for virus conjugated with TMPEG, SSPEG and CCPEG chemistries, respectively [80]. Although these assays were able to confirm that virus particles were modified once the PEGylation process was complete, they did not accurately assess the degree of modification.

**Figure 6.**
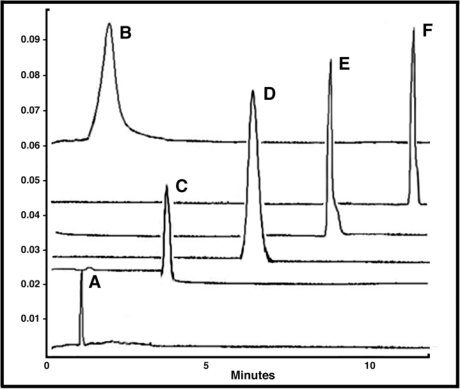
PEGylation Significantly Alters the Surface Charge of the Adenovirus Over Time. Representative Capillary Electropherograms of **(A)** Monomethoxypoly(ethylene) glycol tresylate (TMPEG, 10 mg/ml) alone and adenovirus that underwent conjugation for **(B)** 24 hours (100% coverage as determined by a fluorescamine assay) **(C)** 4 hours (90% coverage) **(D)** 2 hours (70% coverage) **(E)** 1 hour (50% coverage) and **(F)** Unmodified Virus. As seen from the figure, capillary electrophoresis can be used to (a) confirm that free PEG has been adequately removed from a preparation, (b) assess the degree of modification of virus capsids and (c) assess the homogeneity of a preparation (*i.e.,* all capsids are modified to the same degree). Data included in the figure was generated by diluting samples 1:2 with sample buffer (20 mM sodium phosphate, pH, 7.0, 5 mM NaCl). Capillary length was 34 cm. Virus was detected at 214 nm. The Y axis represents absorbance units and the X axis minutes until a preparation eluted from the capillary.

**Figure 7.**
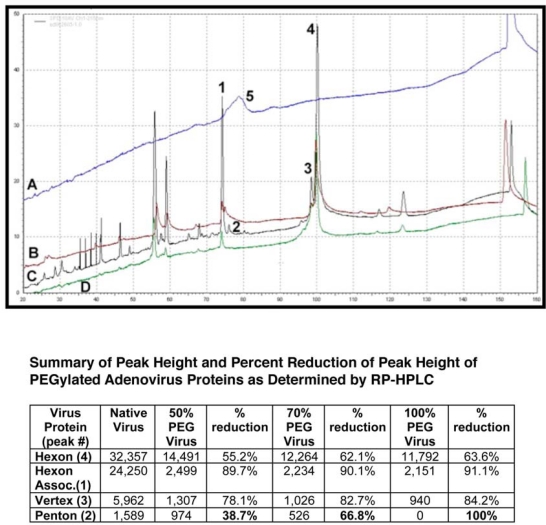
PEGylation Dampens Peak Intensity of Adenovirus Capsid Proteins as Determined by Reverse Phase HPLC. RP-HPLC Chromatograms Showing Peaks of **(A)** Free PEG; **(B)** PEGylated adenovirus (50% modification as determined by CE and fluorescamine assays); **(C)** Unmodified Adenovirus and **(D)** PEGylated adenovirus (100% modification). Viral proteins were separated on a Jupiter column (250 × 4 mm) packed with a 5 μm diameter, 300 Å pore size C4 resin (Phenomenex) and a pre-column filter (0.5 μm, Phenomenex) at 45 °C. A 145 minute gradient of 0.1% trifluroacetic acid (TFA) in water (Solution A) and 0.1% TFA in acetonitrile (Solution B) was used at a flow rate of 1 ml/min and absorbance measured at 215 nm. Reduction of the peak height of the penton protein (Peak 2) reflects the degree of modification of the virus capsid as determined by fluorescamine and biotin ELISA assays (see table for data summary). This method can also be used to monitor the PEGylation process and confirm results obtained from analysis by capillary electrophoresis (Figure 6). It also verifies that free PEG is removed from the final preparation as is shown by the absence of Peak 5 in all traces.

**Figure 8.**
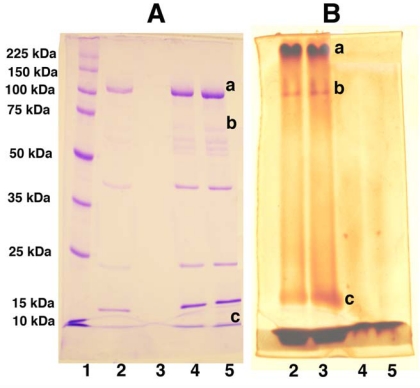
Changes in the Molecular Weight of PEGylated Adenovirus Capsid Proteins Can be Detected by Gel Electrophoresis and Barium Iodide Staining. Unmodified (Lanes 4 and 5 both gels) and PEGylated (Lanes 2 and 3 both gels) adenovirus were boiled and run on 10% polyacrylamide gels with standard molecular weight markers (Lane 1 gel A) at 30 volts overnight. Duplicate samples were run such that, when electrophoresis was complete, the gel could be cut in half and either stained with Coomassie Brilliant Blue (Gel A) or a 5% barium chloride/1M iodine in 0.01 M perchloric acid (Gel B) for the identification of PEGylated proteins as described in reference 88. PEGylated proteins (Lanes 2 and 3, Gel A) are not stained as intensely as unmodified proteins (Lanes 4 and 5, Gel A) with Coomassie Blue despite the fact that the same amount of virus (based upon protein concentration) was loaded in each lane. In contrast, unmodified proteins were not resolved with barium chloride/iodide staining (Lanes 4 and 5, Gel B) while proteins with high PEG densities (Lanes 2 and 3, Gel B) could be detected by this method. Changes in the molecular weight of the adenovirus hexon (marked “a”), penton (marked “b”) and hexon-associated protein (marked “c”) were noted in the preparation included in the figure. The limit of detection of this assay was 0.5 μg of PEG in a 10% acrylamide gel.

Changes in particle size of the virus have been found to correlate with degree of modification. In one report, addition of 5 kDa PEG to all of the available sites on the adenovirus capsid increased the diameter of the particle by approximately 30% [86]. Other efforts have focused upon assessment of the number of unmodified amino groups that remain on virus prior to and after PEGylation process using a traditional fluorescamine assay in which measured fluorescence is proportional to the free lysine amino groups on the virus capsid [80] or a PEG-biotin enzyme-linked immunosorbent assay (ELISA) that indirectly quantifies the amount of biotin-labeled PEG associated with a virus particle using an avidin-horseradish peroxidase detection system [79]. Pairing these assays with other analytical techniques such as capillary zone electrophoresis and high pressure liquid chromatography (HPLC) where subtle changes in physical properties of individual capsid proteins can be measured by changes in the placement of a peak and/or peak size (Figures 6 and 7) and sodium dodecyl sulfate polyacrylamide gel electrophoresis (SDS-PAGE) with barium iodide staining that allows one to visualize specific virus proteins linked to PEG and measure the change in molecular weight associated with the new PEG-conjugate [88] (Figure 8) will improve the accuracy of the chemical profiles of PEGylated viruses and assist in the assessment of the reproducibility and reliability of a PEGylation process prior to clinical testing.

### 4.5. Pharmacology of Adenovirus-Pharmacokinetics and Biodistribution

As mentioned previously, one of the benefits of PEGylating therapeutic proteins is that this modification prolongs the circulation time, allowing for administration of a single daily dose to achieve full therapeutic effect. When given systemically, the half-life unmodified adenovirus is less than 2 minutes [89]. The kinetics of PEGylated adenovirus were initially described by Alemany *et al.* who indicated that the clearance rate of the modified virus is reduced by a factor of 4 with respect to unmodified virus in mice [89]. Similarly, it has been reported that PEGylation reduces the systemic clearance of helper-dependent adenovirus by approximately 40% in non-human primates [90]. The circulation time of a PEGylated adenovirus targeted to endothelial cells in the blood was found to be significantly longer than that of unmodified virus after systemic administration in mice, as evidenced by an increase in the area under the plasma concentration-time curve (AUC) by a factor of 12 [91]. The half-life of PEGylated adenovirus also increases profoundly with the degree of modification (Table 4) [86]. Plasma half-lives of adenoviruses linked to 5 kDa PEG at a ratio of 90% and 100% were 6.4 and 22 min, respectively, whereas that of unmodified Ad had a calculated half life of 1.9 min in mice. Increasing the molecular weight of the PEG also prolongs the time that the virus remains in the systemic circulation (Table 4). Plasma levels of adenovirus conjugated with 20 kDa PEG at a 45% modification ratio were 25 times that of unmodified virus or 8 tines that of virus coupled with 5 kDa PEG at a 90% modification ratio 60 minutes after intravenous administration in mice [92]. Similarly, it was reported that circulating genome levels of virus modified with 35 kDa PEG were more than 10 fold higher than those of unmodified virus at both early (10 minutes) and later (24 hours) times after systemic administration in mice [93].

**Figure 9.**
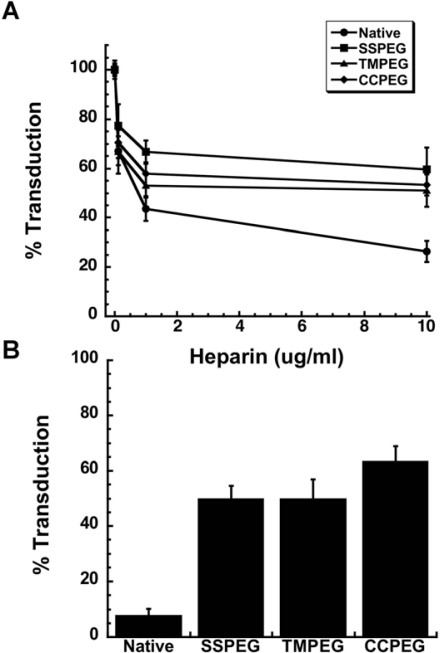
**(A)** Effect of Heparin on Adenovirus Transduction Efficiency. Viruses were pre-incubated with heparin for 1 hour at 37 °C prior to addition to A549 cells. Virus binding was allowed to take place for 1 hour at 4 °C prior to the replacement of virus with culture medium. Values are representative of three separate experiments. Error bars represent the standard deviation of the data; **(B)** PEGylated Adenoviruses Enter Target Cells Partially Through Heparan Sulfate Glycosaminoglycans. Virus was pre-incubated with heparin (10 μg/ml) prior to addition to monolayers of A549 cells treated with both the anti-CAR antibody and a peptide which blocks integrin receptors (RmcB + RGD + Hep.). Percent transduction is the number of beta-galactosidase positive cells found in treated monolayers with respect to the number of positive cells found in monolayers that did not receive treatment prior to viral infection. Data are the average transduction efficiencies obtained from two separate experiments and error bars represent the standard deviation of the data.

Removal of adenovirus from bloodstream and rapid uptake in the liver after intravenous injection is caused by non-specific charge-mediated interactions with proteins and cells, notably components of the reticuloendothelial system (RES) [89]. It is also known that adenovirus preferentially accumulates in the liver and the spleen in rodent models and non-human primates when given systemically, which contributes significantly to the toxicity associated with this vector [94–96]. Although the exact mechanism by which PEG prolongs circulation time in the blood is not currently known, it is thought that protrusion of long chain PEG molecules from the virus surface plays a critical role in minimizing cellular interactions, limiting access to the liver parenchyma and subsequently extending circulation half-life [84,97,98]. If this is indeed the manner by which PEGylation extends the half–life of the virus, one must also reason that transduction efficiency is also compromised. This has been reported *in vitro* [84], however, we and others have found that conjugation of virus with 5 kDa PEG does not compromise hepatic transduction efficiency of both first generation and helper dependent adenoviruses in mice [82,84,93,99]. One reason for this may be the longer circulation time, which prolongs virus contact with the liver. In addition, *in vitro* data (Figure 9, unpublished data) and studies in rodents have suggested that PEGylated virus retains its liver transduction efficiency by entering cells via heparan sulfate proteoglycan receptors (HSPGRs), the primary means by which the virus enters hepatocytes *in vivo*, and interacts with blood factors including protein C (PC), factor VII (FVII), factor IX (FIX) and factor X (FX) which facilitate binding of virus to these receptors [93,100–105].

A recent study in which it was reported that PEGylation reduces the transduction efficiency of helper-dependent adenovirus in the liver of non-human primates suggests that data generated in rodent models may not predict how these modified viruses will perform in the clinic [90]. Although the mechanism(s) underlying the differences in hepatic transduction efficiency of PEGylated virus between these species are unclear, species-specific differences in liver fenestration and in the binding properties of platelets and other blood components with the modified virus may attribute to this finding in non-human primates. Although the average diameter of fenestrae of a mouse and a baboon are similar (100 and 90 nm, respectively), the number of fenestrae in the baboon liver is less than that of the mouse by a factor of 10 [106]. Thus, the smaller size and lower density of fenestrae may be anatomical barriers for hepatocyte transduction in non-human primates. This may also explain reduced hepatic transduction efficiency of virus modified by high molecular weight PEGs at high PEG densities reported in mice [86,92,93].

### 4.6. Toxicology of PEGylated Adenovirus

Adenovirus-induced toxicity, caused by the immune response to virus capsid proteins, occurs shortly after systemic administration in several animal models and humans [76,107–110]. Covalent attachment of PEG to the virus capsid significantly alleviates virus-induced hepatotoxicity and cytotoxicity in mice. Serum alanine aminotransferase (ALT), an indicator of hepatotoxicity, was reduced in mice given PEGylated first generation and helper-dependent adenovirus by a factor of 13 and 9 with respect to animals given unmodified virus [99]. Similar results were also found for serum aspartate aminotransferase (AST), another indicator of hepatotoxicity in non-human primates [90]. Serum lactate dehydrogenase (LDH), an indicator of tissue and organ damage, also followed a similar trend, further indicating that PEGylation can reduce adenovirus-associated toxicity in non-human primates. While one might reason that these observations are simply due to the fact that the virus does not efficiently transduce the primate liver, additional data from this and other studies suggest that it is a reduction in uptake of the PEGylated virus in the spleen and subsequent drop in cytokine release (see Section 4.7 below) that is responsible for the improved toxicity profile of PEGylated vectors in this animal model.

Abnormalities associated with blood coagulation including prolongation of clotting time, a reduction in platelets, and an increase in D-dimer levels have been observed after administration of adenovirus in several animal models and humans [107,110–113]. A significant drop in platelet counts from 1,644 × 10^3^/μL to 770 × 10^3^/μL was observed in mice at three days after administration of unmodified helper-dependent virus while animals treated with PEGylated virus did not experience a change in platelet counts from baseline levels [99]. Similarly, conjugation of a second generation adenovirus with SSPEG and TMPEG completely prevented thrombocytopenia [114]. Recent studies in mice demonstrated platelet counts were unaffected by administration of PEGylated virus at a dose of 1 × 10^11^ viral genomes (vg)/kg while a slight decrease was detected at a dose of 3×10^11^ vg/kg. In this study, PEGylation also reduced production of D-dimer by a factor of 2.4. Further investigation illustrated that PEGylation reduces the binding of virus to platelets and erythrocytes. Taken together, it was suggested that steric hindrance associated with the polymer prevents interaction with platelets, which in turn, disrupts the clotting cascade and also limits endothelial cell-mediated platelet activation and clearance, leading to reduced D-dimer formation and subsequent thrombocytopenia [115]. These differences in binding may also explain the fact that a 40% reduction in platelets was noted in baboons 6 hours after administration of 3 × 10^12^ vp/kg of both unmodified and PEGylated helper-dependent virus [90]. This transient drop returned to baseline within 72 hours in a baboon given PEGylated virus while platelets remained low throughout the study in an animal given unmodified virus. D-dimer was affected in the same manner.

### 4.7. The Immune Response

Most of the toxicity associated with recombinant adenoviruses arises from the innate and adaptive immune response against the virus and develops in three phases in animal models and humans. The first phase, caused by the interaction of adenovirus capsid with Kupffer cells, macrophages and dendritic cells, occurs as early as 1 hour after systemic administration and continues for four days. This results in the release of pro-inflammatory cytokines and chemokines, including interleukin-6 (IL-6), IL-12, tumor-necrosis factor-α (TNF-α), inducible-protein-10 (IP-10) and RANTES into the general circulation to recruit effector cells, leading to neutrophil-dependent hepatic injury and progression toward the second phase of the inflammatory process [108,116]. This phase, occurring 5 to 7 days after administration, is characterized by removal of vector-infected cells by activated lymphocytes, leading to short-term transgene expression and a self-limited inflammation in the liver [117,118]. At high viral loads, this can progress to severe liver necrosis, disseminated intravascular coagulopathy, bleeding and, in a few cases, a systemic inflammatory response syndrome, a condition characterized by multiorgan failure, sepsis and trauma [76,107]. The last phase, the humoral immune response, is characterized by production of antibodies which rapidly clear the virus from the circulation and prevent successful gene transfer upon readministration [75].

Various strategies have been evaluated to circumvent both cellular and humoral immune responses generated against adenoviral vectors. Immunosuppression with agents such as cyclophosphamide, FK506 and cyclosporin A [119–123] and the disruption of costimulatory interactions between T cells and B cells using interferon-γ, IL-12, anti-CD40 ligand antibody or CTLA4Ig [124–127] have effectively blunted the immune response and extended the length of transgene expression. However, these approaches were deemed unsuitable in practice since they impair immunity to other microbes and produce serious unwanted side effects. Deletion of all viral early and late genes in helper-dependent adenoviruses has reduced cell-mediated immune responses, resulting in high level and long-term transgene expression [128]. However, acute toxicity and neutralizing antibodies are still detected after treatment with this vector [129]. Recent efforts employing genetic substitution of both the fiber, hexon and other capsid proteins with those of rare and non-human adenovirus serotypes have shown to dampen the innate response and subsequent release of inflammatory cytokines [130–133]. Although viruses constructed by this approach, termed “sero-switching”, are minimally affected by anti-adenovirus type 5 neutralizing antibodies and can be successfully given several times, they are very difficult to produce.

O’Riordan *et al.* was the first to show that covalent attachment of a polyethylene glycol to adenovirus capsid proteins was a straightforward and practical strategy to prevent neutralizing antibodies (NAB) from recognizing virus surface antigens [79]. Conjugation of virus with 15% and 20% TMPEG improved *in vitro* transduction efficiency in the presence of neutralizing antibodies 2 fold with respect to unmodified virus. The most striking finding of these studies was that administration of PEGylated virus to mice previously exposed to unmodified virus produced transgene expression levels equivalent to those found in naïve animals. Since then, many other groups reported similar results [81,82,91,97,134–137].

PEGylation has attenuated the acute toxicity associated with the innate immune response by reducing production of pro-inflammatory cytokines and chemokines and infiltration of neutrophils in the liver. Early reports demonstrated that mice treated with PEGylated first generation and helper dependent adenoviruses had serum IL-6 levels that were 4 and 2 times lower respectively than that seen in mice given unmodified virus 6 hours after treatment. At the same timepoint, serum IL-12 in animals treated with PEGylated virus was 3 fold lower than animals treated with unmodified virus. PEGylation has also shown to reduce TNF-α to baseline levels [99]. Further characterization of cytokine release profiles in mice revealed that PEGylated virus produced similar kinetic profiles for IL-6 and other cytokines to that of animals receiving unmodified virus, meaning that levels still peaked at 6 hours [84]. However, PEGylation of first generation and helper-dependent viruses significantly reduced cytokine levels as early as 2 and 4 hours after treatment as well as at 6 hours by a factor of 8, 3 and 5 respectively [114]. Similar to that reported in rodents, IL-6 levels were also found to be reduced by approximately 70% in non-human primates given a single intravenous dose of 3 × 10^12^ vp/kg dose of PEGylated helper-dependent adenovirus [90]. In the same study, IL-12 was reduced by a factor of 2 while TNF-α was not detected in the animal given the PEGylated vector. Real-time RT-PCR analysis indicated that hepatic gene expression of chemokines including monocyte chemo-attractant protein-1 (MCP-1), macrophage inflammatory protein-2 (MIP-2), macrophage inflammatory protein-1β (MIP-1β), interferon-inducible protein-10 (IP-10) and lipopolysaccharide-induced CXC chemokine (LIX) was significantly reduced in mice after tail vein injection of PEGylated virus and that neutrophil infiltration in the livers of these mice was reduced by a factor of 2 with respect to those given unmodified virus [114]. *In vitro* mechanistic studies revealed that macrophages infected with PEGylated virus failed to produce IL-6 [85,138]. Other studies report that vector uptake by macrophages and Kupffer cells *in vitro* and *in vivo* is significantly hindered by PEGylation [84]. Taken together, one may attribute the reduction in immunogenicity inflicted upon the adenovirus by the PEGylation process is due to the fact that the polymer prevents uptake and processing of the virus by antigen presenting cells [84,97,114].

Additional studies with PEGylated adenoviruses revealed that this modification dramatically attenuated cellular immune responses [81,82,97]. Significant reductions in cytotoxic T lymphocyte production were noted after a single intratracheal and intravenous dose of PEGylated virus in mice [81,82]. Modification of virus with PEG alone or PEG and a peptide specific for the fibroblast growth factor receptor reduced secretion of IFN-γ and IL-2, markers of the Th2 response [97]. As might be expected, the transgene expression profiles achieved with several PEGylated vectors were significantly extended beyond what was commonly seen with unmodified viruses [81,82,99]. As seen with native virus, transgene expression after intratracheal instillation of PEGylated virus also peaked in mice at 4 days, but continued for 42 days, long after that from the native virus dwindled (10 days after treatment) [81]. Similarly, transgene expression was extended from 14 to 28 days after a single intravenous injection of PEGylated virus in the mouse [82]. Although the exact mechanism by which PEGylation dampens the T cell response has not been identified, it is likely that the polymer alters processing and presentation of viral antigens, preventing T cells from recognizing viral epitopes. It is also possible that PEG modification redirects the vector away from antigen-presenting cells (APCs). The fact that several reports have described unsuccessful readministration of PEGylated virus in mice treated with virus modified by the same PEG chemistry and that changing the chemistry of the polymer reestablished transgene expression suggests that shielding traditional immunogenic peptide sequences through PEGylation of capsid proteins may induce processing and recognition of other sequences in close association with the polymer [81,82,99]

## 5. Re-Directing Adenovirus by Physical Means: Effect of Molecular Size and Degree of PEGylation

Another major obstacle to clinical use of adenoviruses for gene transfer is the fact that the virus can infect many different cells due to widespread expression of its primary cellular receptors: the coxsackievirus and adenovirus receptor (CAR) [139] and integrins [140,141]. Interestingly, these receptors are either absent or expressed at low levels on the surface of many cells that are logical targets for gene transfer such as tumor, skeletal and smooth muscle, peripheral blood, and hematopoietic stem cells, making gene transfer relatively inefficient [142,143]. One observation made during the characterization of the transduction efficiency of PEGylated vectors was that the both the size of the PEG and the level and the degree of modification could prevent transduction of many tissues and redirect the virus to some of these important targets. Initial studies found that systemic administration of adenovirus conjugated with 5 kDa PEG at a density of 90% increased transgene expression by a factor of 35 in tumor bearing mice and reduced transgene expression in the liver by 7% [86].

Further characterization of PEGylated viruses suggested that vectors modified with large molecular weight polymers would indeed be useful in the treatment of cancer. Several groups found that polymers with sizes in the 2–5 kDa range could not completely ablate the interaction of the virus with integrin receptors allowing for transduction the liver through endothelial cells [84,92,93,137,144]. Use of larger, 20 kDa polymers prevented transduction of both liver after systemic administration and muscle after local injection in mice [137]. In addition, use of this large polymer at a 45% PEG density improved transgene expression by a factor of 5 in tumor bearing mice while reducing gene expression in the liver by a factor of 185 improving the therapeutic window (ratio of gene expression in the tumor to that in the liver) over that seen with a similar virus modified with 5 kDa PEG by a factor of 45 [92]. Similar results were observed with oncolytic adenoviruses[144]. Although the exact mechanism(s) underlying tumor targeting and liver detargeting by PEGylation has not been exactly identified, the large PEG molecules that extend from the virus most likely prevent direct interaction of the virus with hepatocytes. This modification also promotes the enhanced permeability and retention (EPR) effect observed within the tumor vasculature since the large size of the vector and its extended circulation half-life allows more virus to reach and remain in the tumor.

### Re-Directing Adenovirus by Chemical Means: Use of PEG as a “Linker” for Attachment of Receptor-Specific Conjugates

Initial efforts to chemically modify adenovirus capsid proteins to facilitate infection of specific cell types involved coupling a receptor-specific ligand such as folate to anti-adenovirus antibodies and mixing this with the native virus [145]. Alternative approaches involved genetically introducing fiber proteins from other adenovirus serotypes into the vector [146] or incorporating homing ligands into virus capsid [147]. Each of these, however were limited by modest production yields on a large scale and the potential for generating new immune responses to the altered vectors [74,148]. As PEGylation chemistry advanced, bifuntional molecules were evaluated for their ability to link the virus to tissue and cell-specific ligands. The strategy for this approach is summarized in Figure 5.

Coupling a biologically selected peptide to the adenovirus capsid using a bi-functional PEG was first reported by Romanczuk and colleagues [134,149]. Modification of the virus with PEG and a peptide specific for differentiated ciliated airway epithelial cells improved transduction efficiency in this target fourfold. Similar findings were reported with PEGylated vectors conjugated with fibroblast growth factor (FGF) both *in vitro* and *in vivo* [97,150,151]. An adenovirus modified with PEG and FGF2 improved transduction of human ovarian cancer cells *in vitro* by a factor of 10 with respect to unmodified virus [97]. Similar results were observed in *vivo* in tumor-bearing CB-17 SCID mice. Retargeting PEGylated virus with FGF was also found to improve transduction by 10–1,000 fold in murine and human muscle cells *in vitro* and by a factor of 6 in the skeletal muscle of mdx mice [150]. Since RGD is a secondary mediator of adenovirus cell entry, its utility as a homing ligand has been demonstrated by several groups [91,136,152–154]. With respect to unmodified virus, RGD-PEG-Ad showed higher levels of gene expression in both CAR-positive and -negative cells, suggesting that the modification dampened CAR-mediated infection of target cells while enhancing the ability of the virus to infect cells through the integrin pathway. Additional improvements in gene transfer to endothelial cells in mice with delayed-type hypersensitivity and human breast cancer cells have been achieved by coupling anti-E-selectin antibodies and folate and human epidermal growth factor (EGF) to PEGylated virus respectively [85,91,138,155]. In addition to improving cell-specific transduction efficiency, this simple approach also offers several other benefits, as described above, including shielding adenovirus from the immune system, improving the half life and other pharmacokinetic parameters of the virus and minimizing safety concerns arising with respect to using genetically and chemically modified viruses in the clinic. [85,91,97,134,136,138,152,155].

## 6. PEGylated Adenovirus-Based Vaccines

Because of their inherent ability to induce strong innate and adaptive immune responses, adenoviruses have been successfully employed as carriers for recombinant DNA–based vaccines [156–158]. One significant drawback to this, however, is the fact that a significant portion of the human population has marked levels of antibodies to the most commonly used adenovirus, serotype 5 [159,160]. Use of non-human serotypes, such as the chimpanzees adenovirus C7 [161,162], or rare human serotypes such as serotypes 35 or 11 has induced strong immune responses in the face of pre-existing immunity [163–166], however, additional work to address issues associated with the safety and large-scale production of these vectors is in order. As significant data generated with PEGylated viruses demonstrated that the vectors efficiently induced transgene expression in the face of pre-existing immunity[81,82,91,97,134,136,137,152], this technology has also found application with respect to vaccine development.

Early work in this area has shown that oral vaccination with PEGylated adenovirus moderately improves the B-cell mediated immune response against Ebola glycoprotein in both naïve mice and those with pre-existing immunity [167]. Virus modified with high molecular weight PEG has also been shown to induce strong cellular and humoral immune responses against the transgene/antigen in mice previously exposed to unmodified adenovirus [137,168]. In a prime-boost approach, priming with virus modified with 5 kDa PEG enabled unmodified and PEGylated vectors to induce stronger T cell responses against an encoded antigen with respect to that achieved with unmodified virus alone [168]. Although these results are promising, work with PEGylated vectors for vaccine development is clearly in the early stages. The exact mechanisms by which these vectors with a reduced immunogenic profile can induce strong anti-antigenic immune responses are currently unknown.

## 7. The Future of PEGylation of Viruses for Gene Transfer and Vaccine Applications

Since the early studies with PEGylated viruses were initiated in the year 2000, a significant number of reports in the literature have made it clear that covalent attachment of many different forms of poly(ethylene) glycol can significantly attenuate the immune response against the virus capsid and improve transduction efficiency in some tissues alone or with the addition of homing ligands to virus modified with heterofunctional chemistries in both mice and non-human primates. Additional bodies of evidence support the notion that much of the toxicity associated with use of the adenovirus is also eliminated by this modification. Despite all these favorable attributes, very few if any of these chemically modified viruses are under consideration for clinical use. Many pre-clinical studies to date suggest that several variables such as the molecular weight and shape of the polymer as well as the conjugation chemistry profoundly dictate the immunological and pharmacokinetic profile of the adenovirus. Further refinement of PEGylation chemistry to improve both site-specific attachment to the virus capsid and homing ligands will minimize the potential for generating mixtures of viruses with different PEG and ligand to virus ratios. Evaluation and validation of additional highly sensitive assays to characterize PEGylated viruses and large scale PEGylation processes will be vital to ensure that protocols are reproducible and that preparations consist of vectors that are modified to the same degree and are free of extraneous reaction byproducts.

It is also important to realize that there are very few studies that determine the exact mechanism by which PEGylation blunts the anti-adenovirus immune response. While there is some evidence that the polymer prevents interaction with antigen presenting cells, other studies using the modified vectors for vaccination purposes suggest that the immune response against the encoded antigen is due to an increase in the uptake and processing of the vector in antigen presenting cells. In addition, there have been a few reports that suggest there is a slight if somewhat delayed immune response against these modified viruses that prevents repeated dosing. As outlined previously, PEGylation was initially employed as a technique by which to induce immunological tolerance against a given antigen. This has not yet been considered with any of the PEGylated adenoviruses to date and should be evaluated for vectors of which multiple doses are required.

## Figures and Tables

**Table 1.** Therapeutic PEGylated proteins currently marketed in the United States and Europe.

**Parent Molecule**	**Generic name**	**Trade name (company)**	**Size of PEG moiety (kDa)**	**Indication**	**Year of approval**
**Adenosine deaminase**	Pegademase bovine	Adagen^®^ (Enzon)	5	Severe combined immunodeficiency disease (SCID)	1990
**L-asparaginase**	Pegaspargase	Oncaspar^®^ (Enzon)	5	Acute lymphoblastic leukemia	1994
**Interferon α-2b**	Peginterferon α-2b	PegIntron^®^ (Schering-Plough)	12	Hepatitis C	2000
**Interferon α-2a**	Peginterferon α-2a	Pegasys^®^ (Genetech)	40	Hepatitis C	2001
**G-CSF**	Pegfilgrastim	Neulasta^™^ (Amgen)	20	Neutropenia	2002
**hGH**	Pegvisomant	Somavert^™^ (Pfizer Pharmacia)	5	Acromegaly	2003
**Erythropoietin**	Methoxy polyethylene glycol-epoetin beta	Mircera (Roche)	40	Anemia	2007
**Anti-TNFα Fab**	Certolizumab pegol	Cimzia (UCB)	40	Rheumatoid arthritis and Crohn’s disease	2008

**Table 2.** Pharmacokinetics of unmodified interferon alpha and two PEGylated derivatives.

**Pharmacokinetic Parameter**	**IFN-α**	**PegIntron (PEG-IFN α-2b)**	**Pegasys (PEG-IFN α-2a)**
Elimination half life (hours)	6–9	32–40	72–96
Clearance (ml/hour)	6,000	725	60–100
Volume of distribution (L)	25–30	20–40	8
Tmax (hours)	7–12	20	80

**Table 3.** Current Summary of PEGylated Viruses and the Reported Impact of Modification.

**Virus**	**Model**	**Biological effects**	**Ref.**
**Adeno-associated virus**	*in vitro*	Conjugation of AAV with monomethoxy poly(ethylene) glycols activated by tresyl chloride (TMPEG) and succinimidyl succinate (SSPEG) chemistries did not compromise transduction efficiency.	66
		PEGylation with either 2 kDa or 5 kDa PEG at 1:1, 10:1, 100:1 and 1000:1 PEG:lysine ratios did not compromise transduction efficiency. Conjugation of rAAV with 2 kDa PEG at the 1000:1 PEG lysine ratio protected from serum neutralization.	67
	*in vivo*	SSPEG and TMPEG improved gene transduction in the lung without compromising transduction efficiency in the liver and muscle. TMPEG reduced Th1-type response. Successful readministration of virus after iv injection was achieved by modification with TMPEG.	66
**Lentivirus**	*in vitro*	Transduction efficiency of PEGylated virus was not compromised in the presence of neutralizing antibodies PEGylation provided a 20 fold resistance to antiserum and extended circulatory half-life by a factor of 5 with no observable loss in titer and prevented interaction with antibodies and inactivation of virus by complement in human and mouse sera.	68
	*in vivo*	PEGylation extended the circulation half-life by a factor of 5 PEGylation improved transduction efficiency in the bone marrow and in the spleen 14 days after systemic administration of virus.	68
**Retrovirus**	*in vitro*	Coating of retrovirus with PEG-poly(L-lysine)(PLL) block copolymer improved transduction efficiency 3 to 7 fold without increasing cytotoxicity.	69
		Conjugation of a (1,2-distearoyl-sn-glycero-3-phosphoethanolamine), polyethylene glycol and biotin complex[DSPE-PEG-biotin] increased the number of viruses that bound to streptavidin coated plates by more than three-fold.	70
**Baculovirus**	*in vitro*	Transduction efficiency was deceased with an increased amount of PEG added to the virus surface.	71
	*in vivo*	PEGylation improved transduction efficiency in the lung and brain.	71
**Influenza virosomes**	*in vitro*	Reconstituted viral membranes containing 3 mol% poly(ethylene – glycol) grafted phosphatidylethanolamine retained 40% of their fusion activity.	72, 73

**Table 4.** Summary of the Reported Impact of PEGylation on the Pharmacokinetic Parameters of Adenovirus In the Mouse.

	**↑AUC**	**↓CL**	**↑Half-life**				
**PEG size**	3.4 kDa	5 kDa	5 kDa				20 kDa
**Degree of modification**	-	-	30%	60%	90%	100%	45%
**Fold change in Pharmacokinetic parameter**	12	4	1.3	2	3	22	25
